# Supplementary material for: Nutritional Analysis of the Spanish Population: A New Approach Using Public Data on Consumption
Source: Int J Environ Res Public Health. 2023 Jan 16;20(2):1642. doi: 10.3390/ijerph20021642 (PMC9867222; doi:10.3390/ijerph20021642)
Supplement: Supplementary file 1 [file ijerph-20-01642-s001.zip › ijerph-2104109-supplementary.pdf]

Supplementary Material (SM)

Extension of methodological aspects

Figure S1: Flowchart of procedures summarized

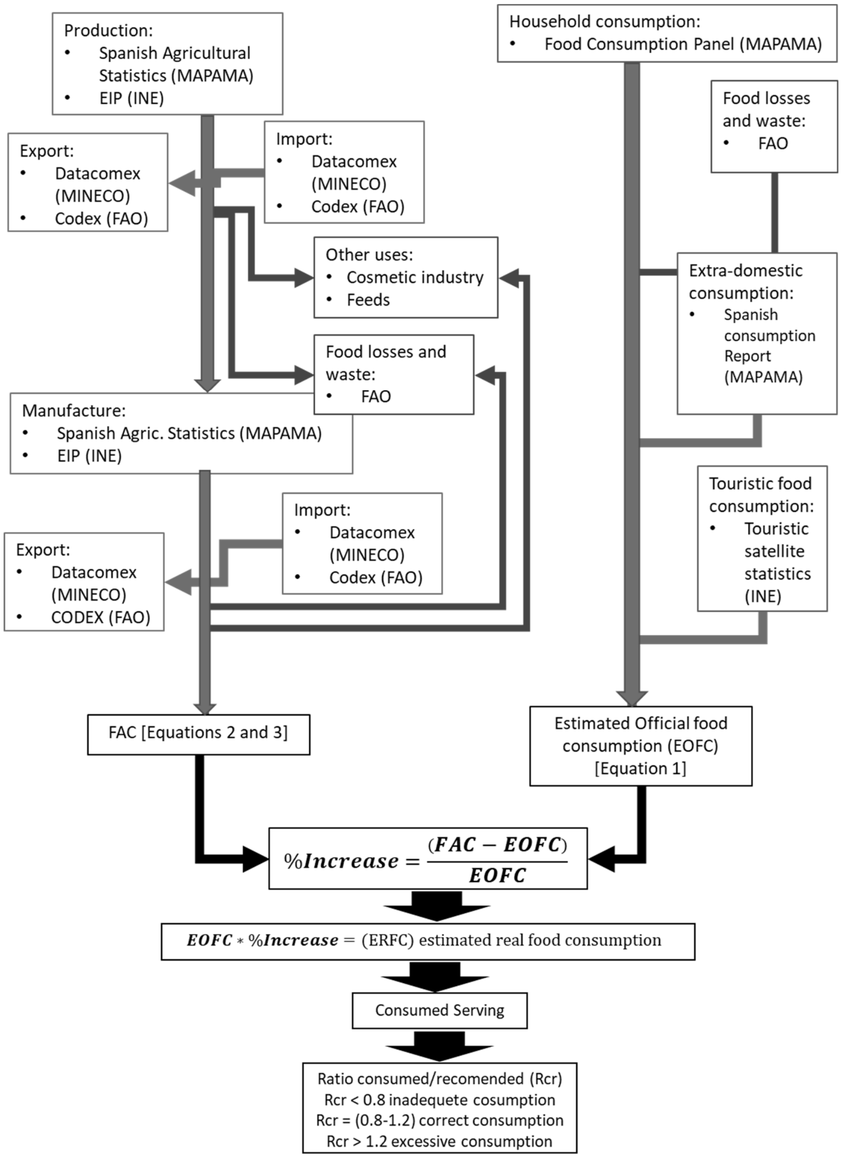

MAPAMA: Spanish acronym of Ministry of Agriculture, Fishing, Food and Environment

EIP (INE): Industrial Products Survey of the National Statistics Institute (INE).

CODEX (FAO): Codex Alimentarius—Food and Agriculture Organization (FAO)

Datacomex (MINECO): Trade Statistics of the Ministry of Industry, Trade and Tourism

Tourist food consumption

In order to know if the tourist demand, in a country like Spain with a high number of visits, can alter the calculations, the consumption of the tourists who visit the country has been estimated. To do this, the duration of tourists' stays has been calculated (INE, 2017a [1]), expressed in number of days and has been multiplied by the three meals eaten per day, according to the average food consumption outside the home. In this way, the total number of meals eaten by tourists has been found. Subsequently, the same procedure has been followed with Spanish tourists who are absent from the country, following the same dietary guidelines shown in the report on non-domestic consumption mentioned above. The balance between the annual number of Spanish visitors and tourists visiting abroad shows a value of less than 3% of total consumption, so it has not been considered necessary to correct the result, as it falls within non-significant margins of error.

Agrarian production

Food loses and waste are not applied to the production phase due to the fact that agrarian production statistics already present net production data.

Disaggregated Spanish industrial production data

For the industrial production of processed products, the Spanish National Institute collects data in its Industrial Production Survey (EIP) (INE, 2017b p27 [2])), presenting a wide range of products. Covering 90% of the production of each code of National Classification of Economic Activities (CNAE) (CNAE 2009: National Economic Activity Classification, homogenized with statistic proposes for the European Union), both in value and volume, the database takes into consideration all companies with more than 20 workers, and more than 50% of companies with fewer workers.

Raw materials and processed products conversion

For the industrial processing of food, we identify the main phases and transformation coefficients in order to identify input quantity need. Therefore, the reconstruction process of the entire tomato industry is explained as an example of any given food chain studied in this contribution:

Tomato transformation processes involve peeling, crushing, and/or concentrating to generate the semi-processed raw materials with which the processed products for consumption are manufactured. As an approximation to the standards of industrial transformation processes, BRIX equivalence graduation has been used. BRIX degrees specify the percentage of solids in the sample and is measured through a refractometric index (Johnstone, Hartz, LeStrange, Nunez, & Miyao, 2005 [3]). A higher value of BRIX degrees corresponds to a greater water evaporation processing of the tomato. According to the CODEX international trade regulation standard for processed tomato, two categories are distinguished: tomato puree (hereafter, tomato concentrate), which contains between 7-24% of total soluble solids (or Brix degrees), and tomato paste, which contains higher values. Starting from the BRIX value contented in fresh industrial tomatoes (Ciruelos Calvo, de la Torre Carreras, & González Ramos, 2008 [4]; Domínguez Pérez et al., 2012 [5]; Lorente et al., 2011 [6]) and the CODEX definitions, we determined the amount necessary for the production of each of the categories included in the Industrial Production Survey (EIP) for the year 2017, therefore, establishing the tons of raw material needed by the manufacturing industries. For some cases, these factors have been corrected based on the information regarding processed tomato products collected from Agribalyse (Asselin-Balençon et al., 2020 [7]) and Ecoinvent 3.7.1 (Ecoinvent, 2019 [13]), as well as from certain literature (Brodt, Kramer, Kendall, & Feenstra, 2013 [8]; Caldeira, De Laurentiis, Corrado, van Holsteijn, & Sala,

2019 [9]; Guzmán Casado et al., 2014 [10]; Secondi, Principato, Ruini, & Guidi, 2019 [11]). Finally, business information sources from the sector or from the brands themselves have been taken into account for the needs of semi-transformed products (tomato paste and tomato concentrate) in the manufacture of final processed products (www.tomcoex.com/es).

Table S1. Conversion factors to tomato equivalent based on BRIX graduation calculus

Product	Conversion factor to tomato equivalent	Group
Fresh tomato	100%	Fresh tomato
Tomato derivative	168%	Processed tomato
Tomato juice	132%	
Ketchup and other tomato sauces	182%	
**Tomato paste (industrial input)**	256%	
**Tomato concentrate (industrial input)**	340%	
Dice or smashed tomato	132%	

Losses associated with production have been applied according to the FAO post-harvest, handling and storage category (FAO, 2011 [12]).

Foreign trade and agricultural and industrial production correspondence

The Foreign Trade Data Statistic (COMEX) collects commodity flows greater than EUR 400,000 of all international trade products, indicating countries of origin and provinces of destination, as well as the main mode of transport used. For the correspondence between the data reflected by the EIP and those of COMEX, we use the document provided by Eurostat (Eurostat, 2019 [14]) to define the correspondence between manufactured products in Spain and those traded with other countries.

Losses associated with post-harvest, handling, and storage have been applied to import flows base on FAO factors (FAO, 2011 [12]). In the case of exports, the value reflected by the COMEX statistics has been escalated to incorporate losses during the agri-exportation flow.

FOOD CHAIN RECONSTRUCTIONS

Figures S2–S21 represent the biomass flows of each food chain used as models for the calculations for the mismatching between official calculated consumption and apparent consumption.

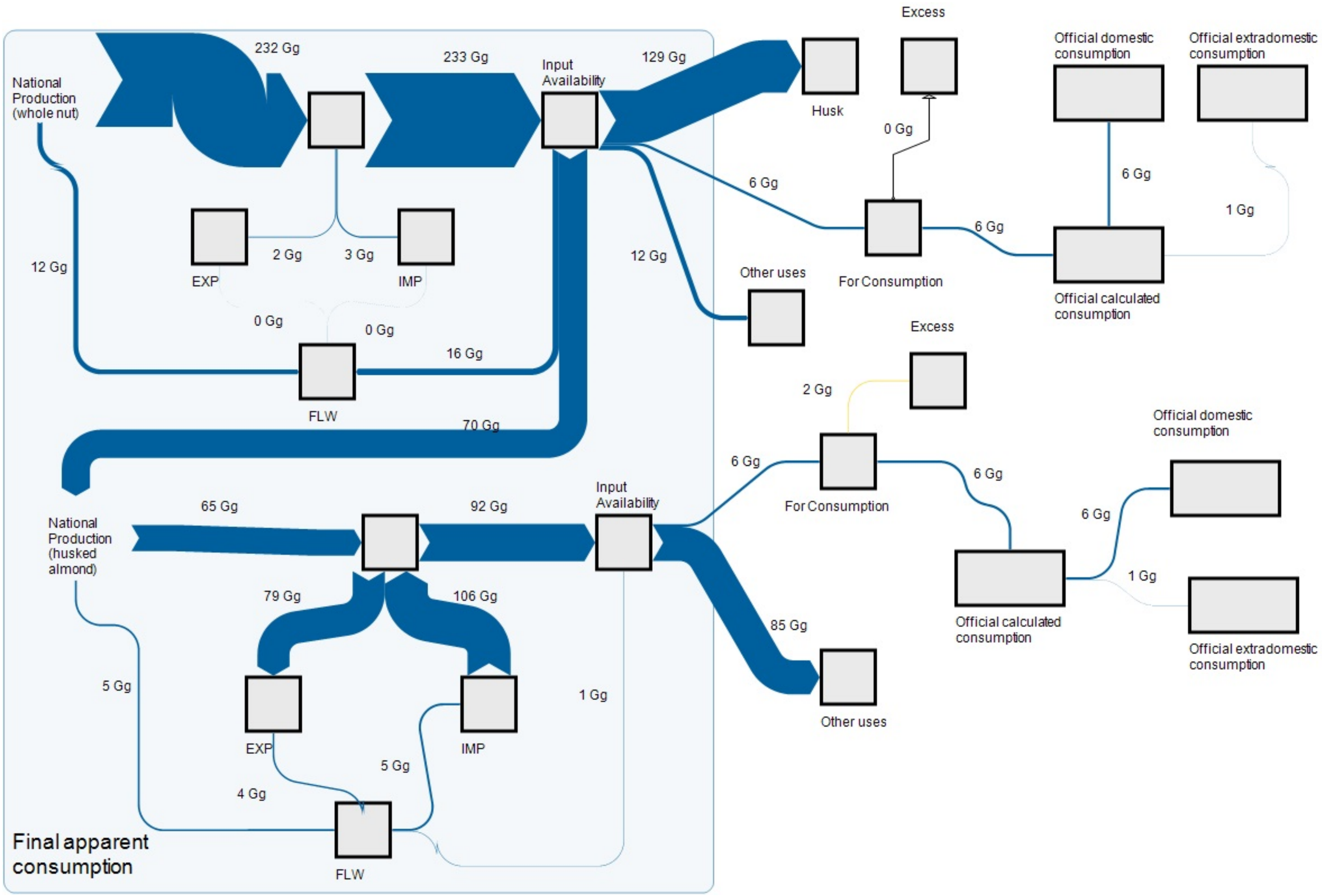

Figure S2 –Diagram of biomass flows for the almond food chain.

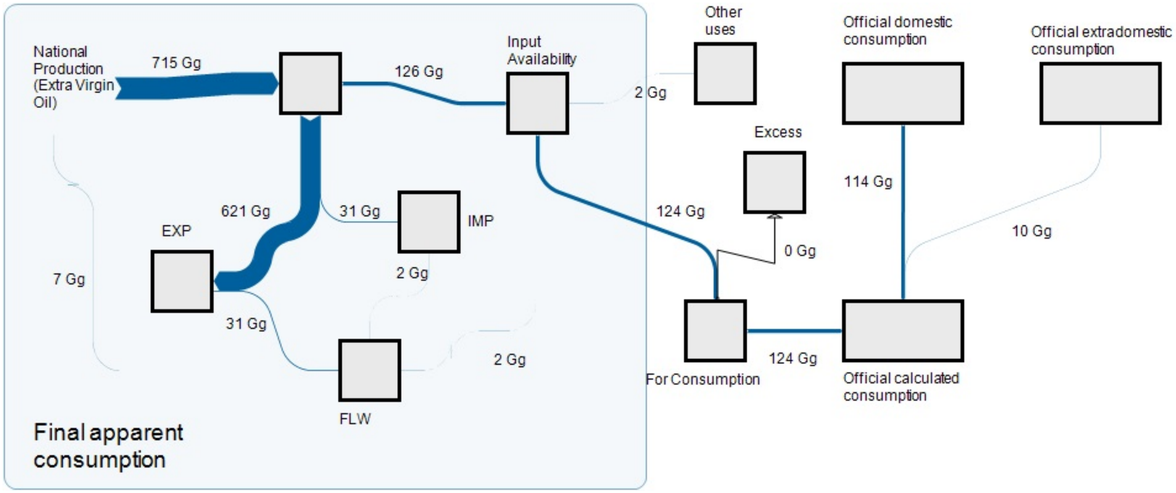

Figure S3 –Diagram of biomass flows for the Extra Virgin Olive Oil food chain.

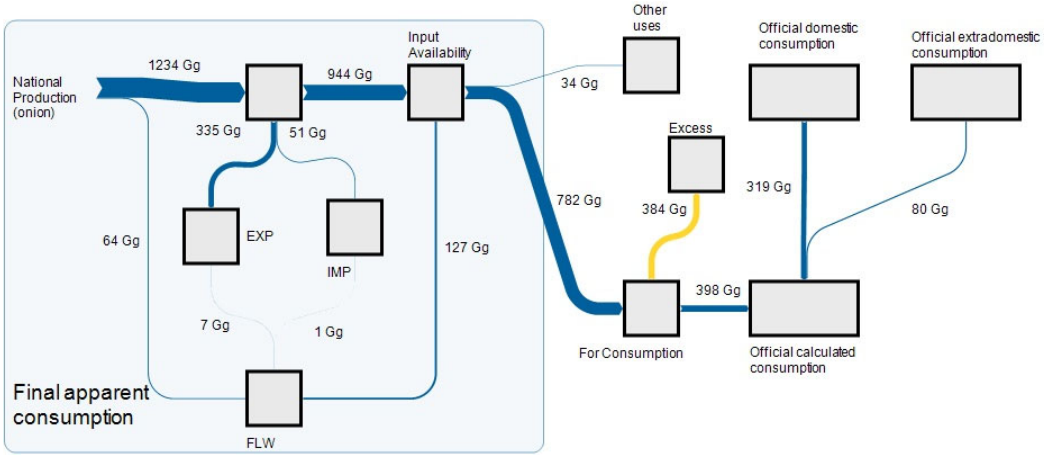

Figure S4 –Diagram of biomass flows for the onion food chain.

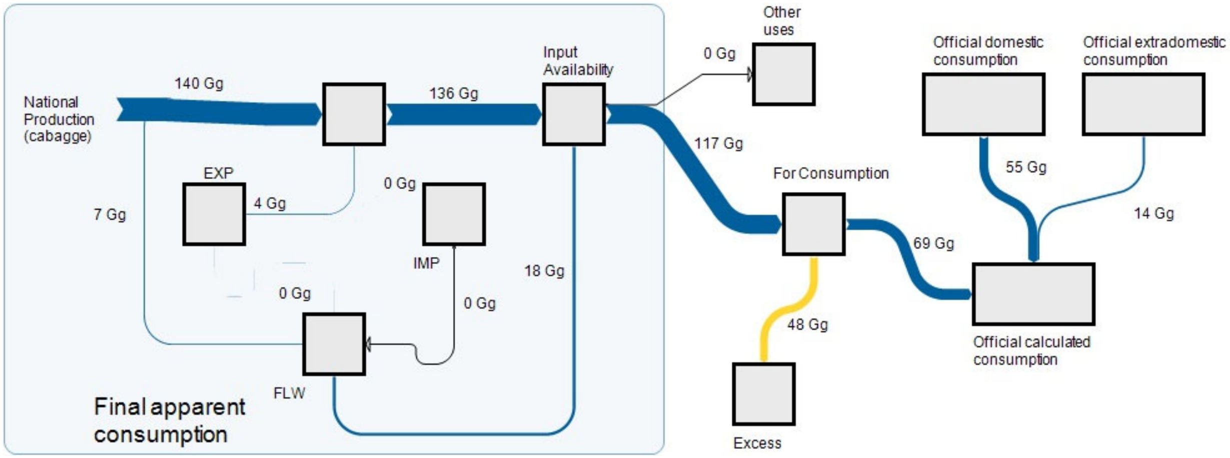

Figure S5 –Diagram of biomass flows for the cabbage food chain.

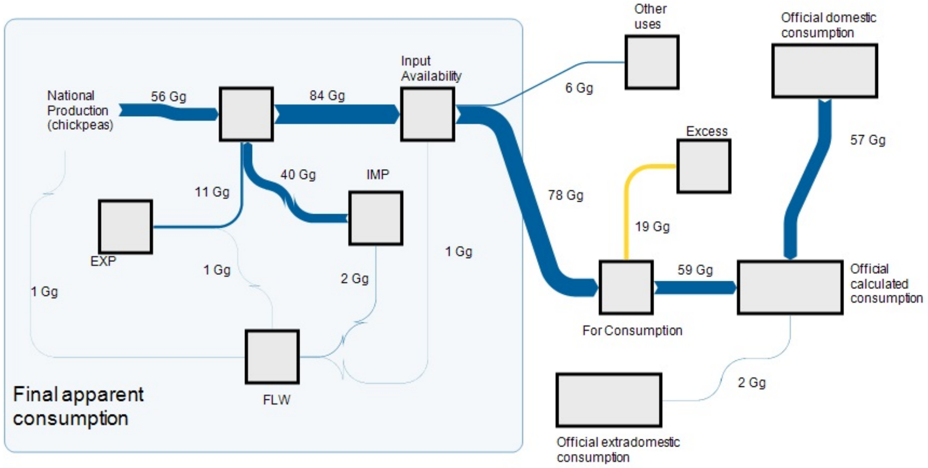

Figure S6 –Diagram of biomass flows for the chickpea food chain.

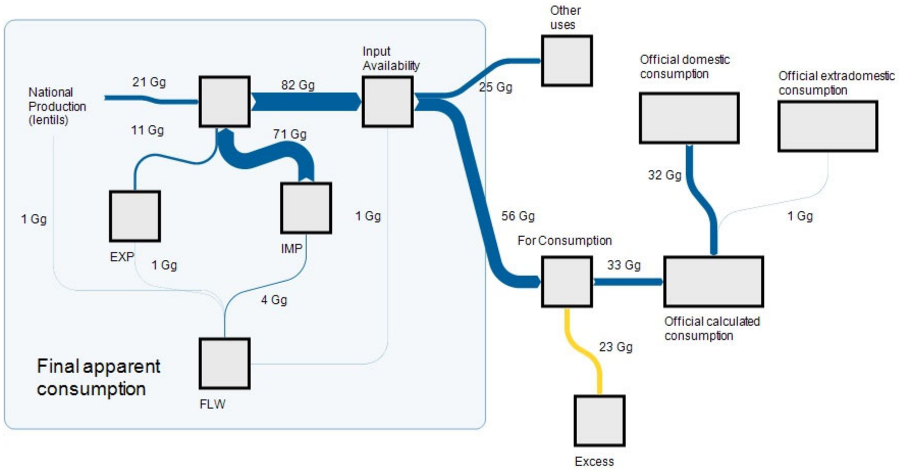

Figure S7 –Diagram of biomass flows for the lentils food chain.

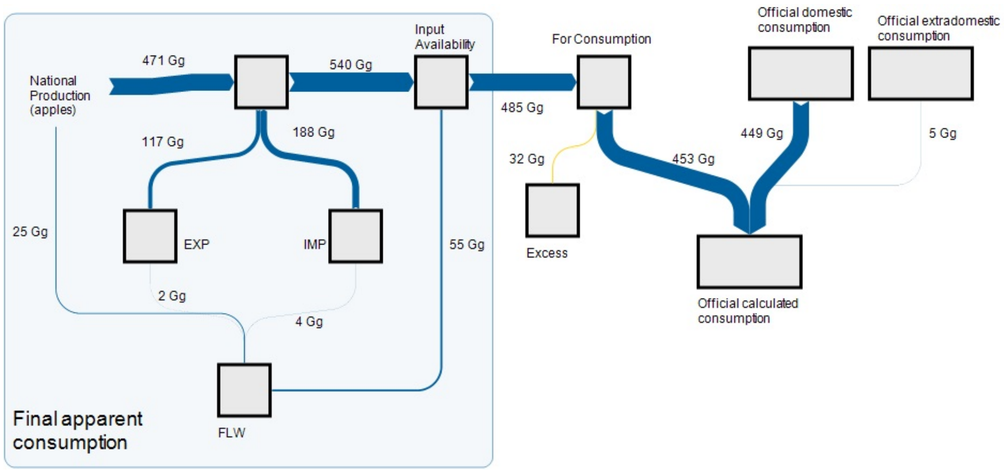

Figure S8 –Diagram of biomass flows for the apple food chain.

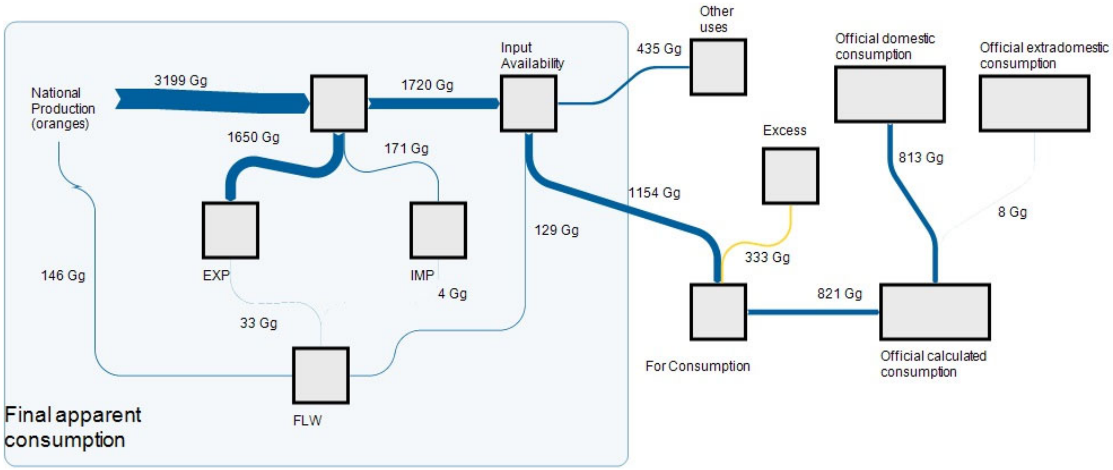

Figure S9 –Diagram of biomass flows for the orange food chain.

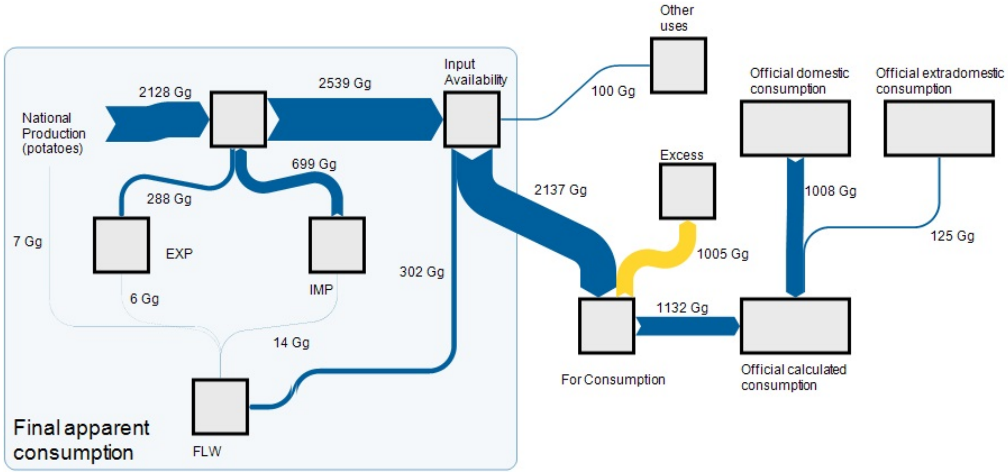

Figure S10 –Diagram of biomass flows for the potato food chain.

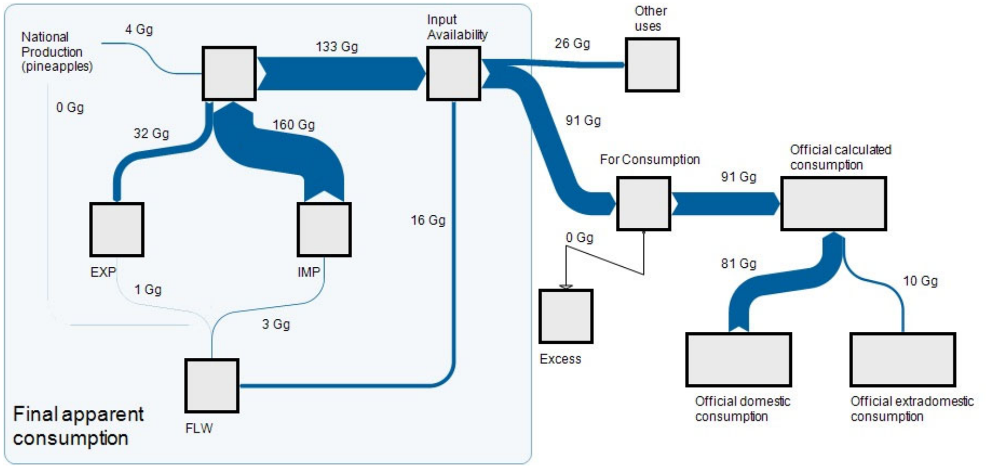

Figure S11 –Diagram of biomass flows for the pineapple food chain.

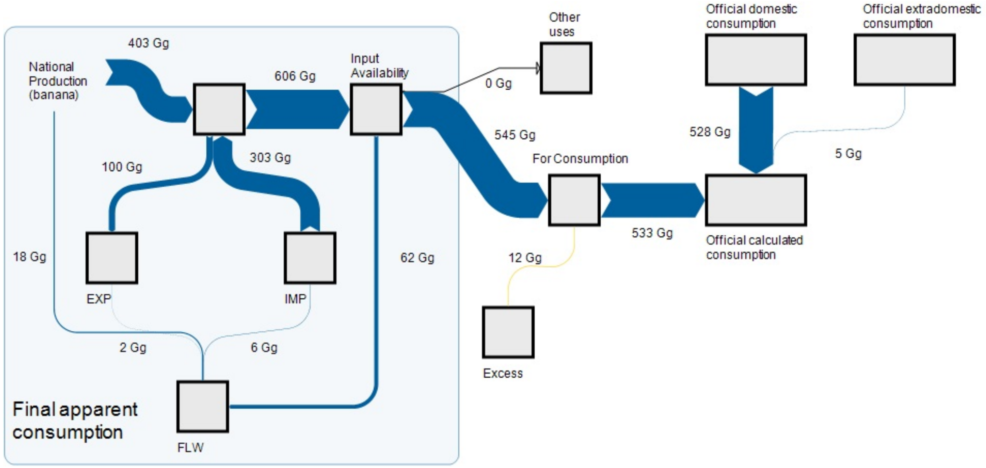

Figure S12 –Diagram of biomass flows for the banana food chain.

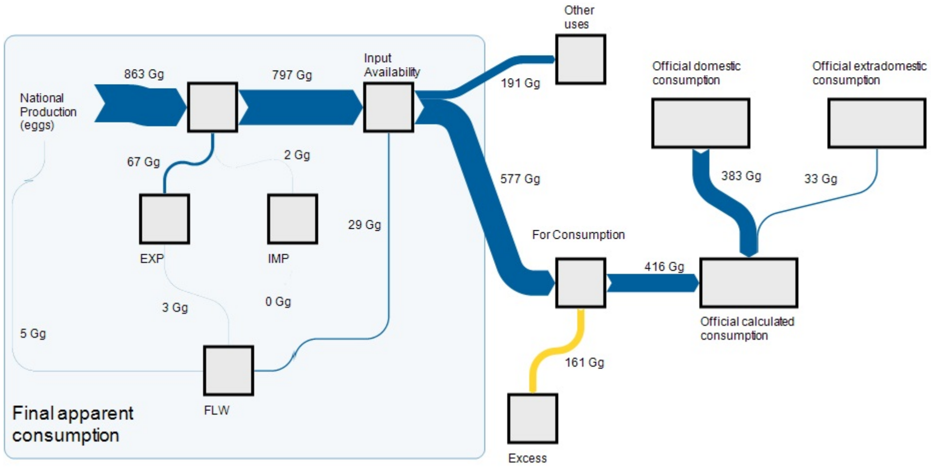

Figure S13 –Diagram of biomass flows for the egg food chain.

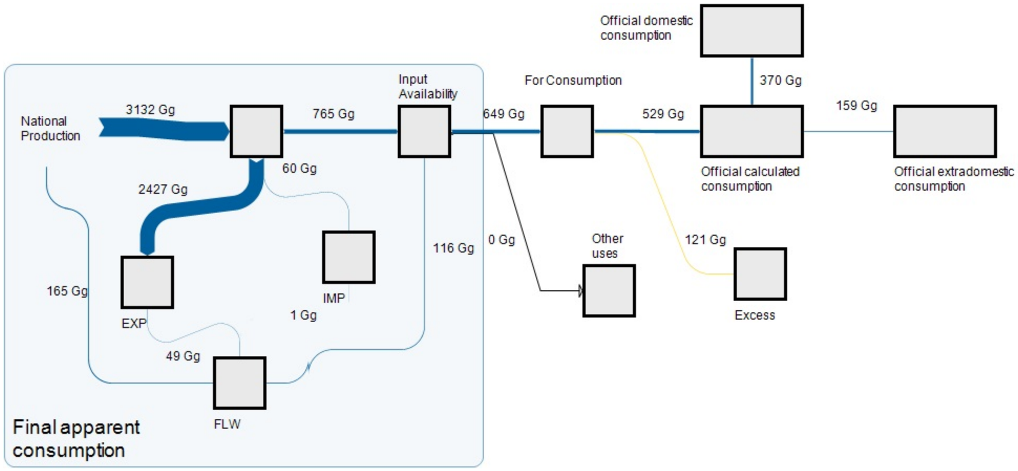

Figure S14 –Diagram of biomass flows for the wine food chain.

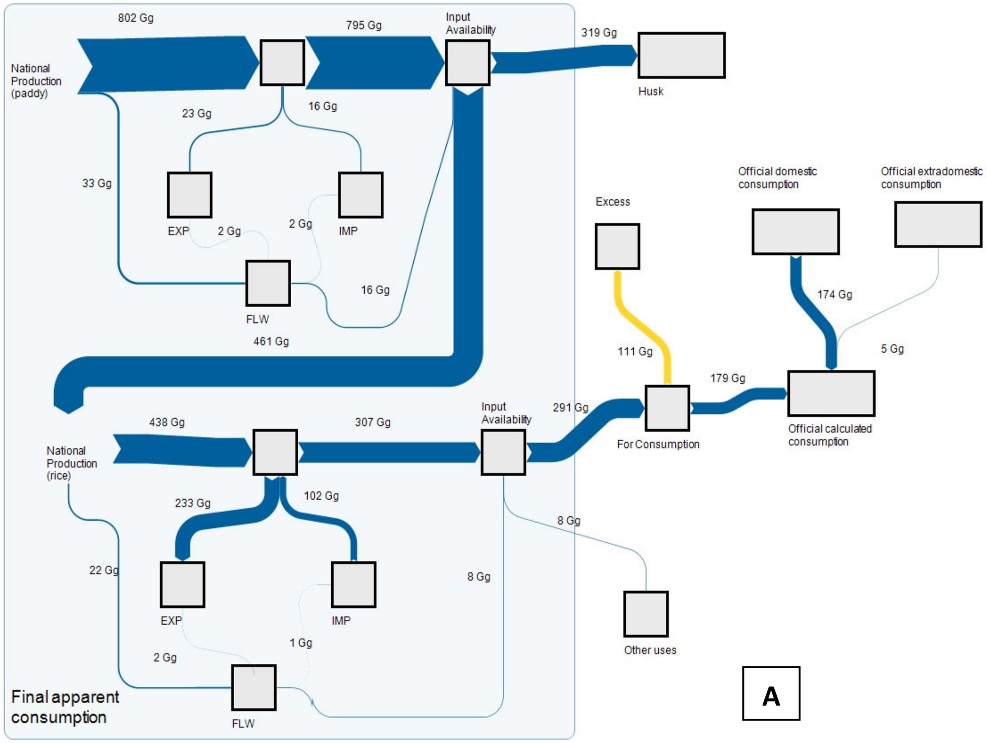

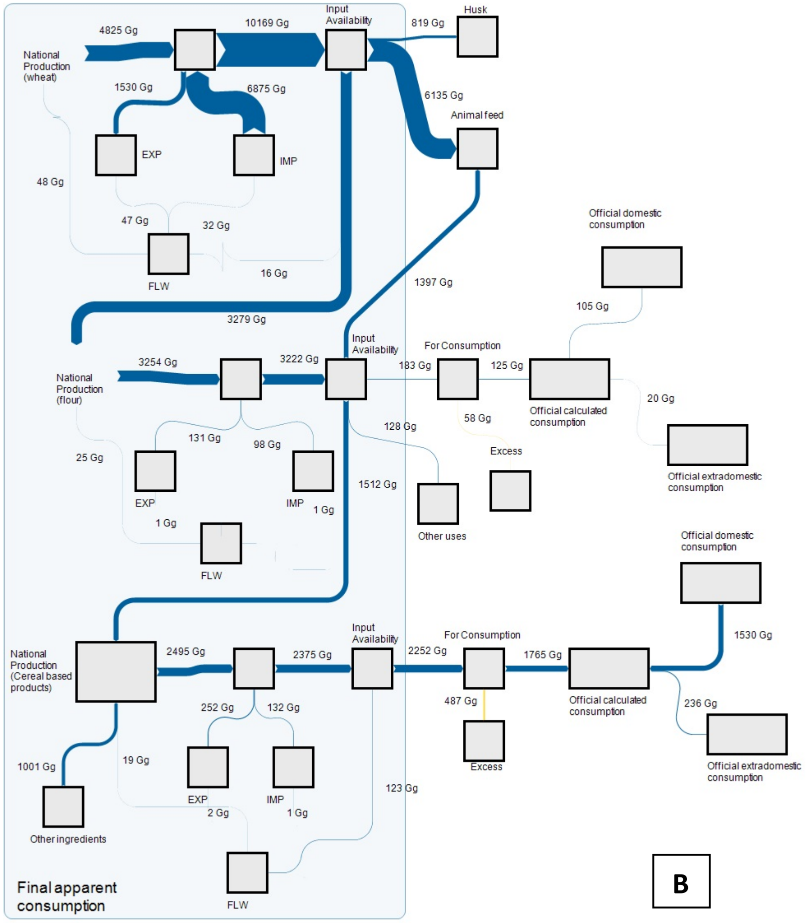

Figure S15 –Diagram of biomass flows for the rice food chain (A) and the wheat food chain (B).

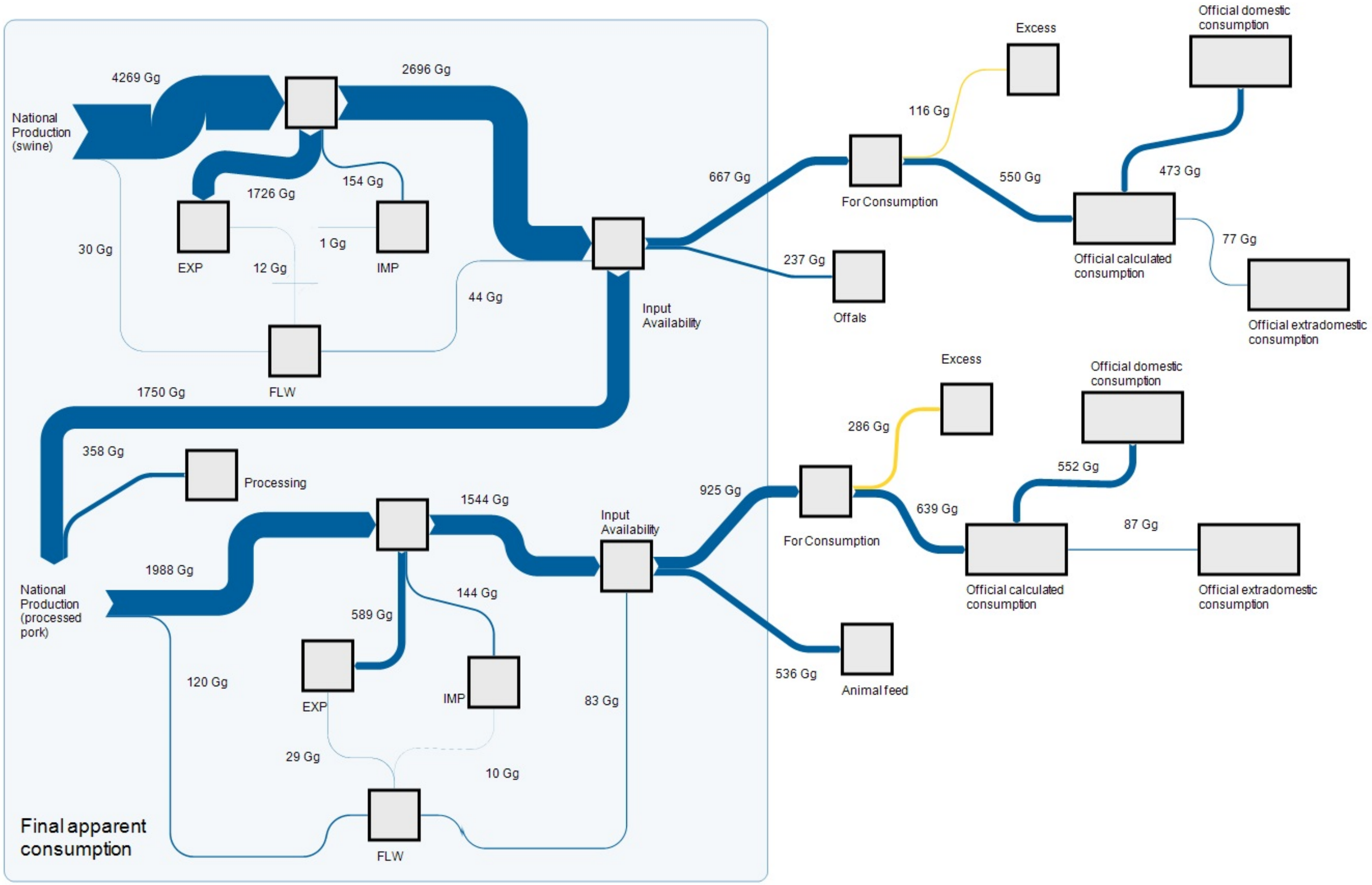

Figure S16 –Diagram of biomass flows for the swine food chain

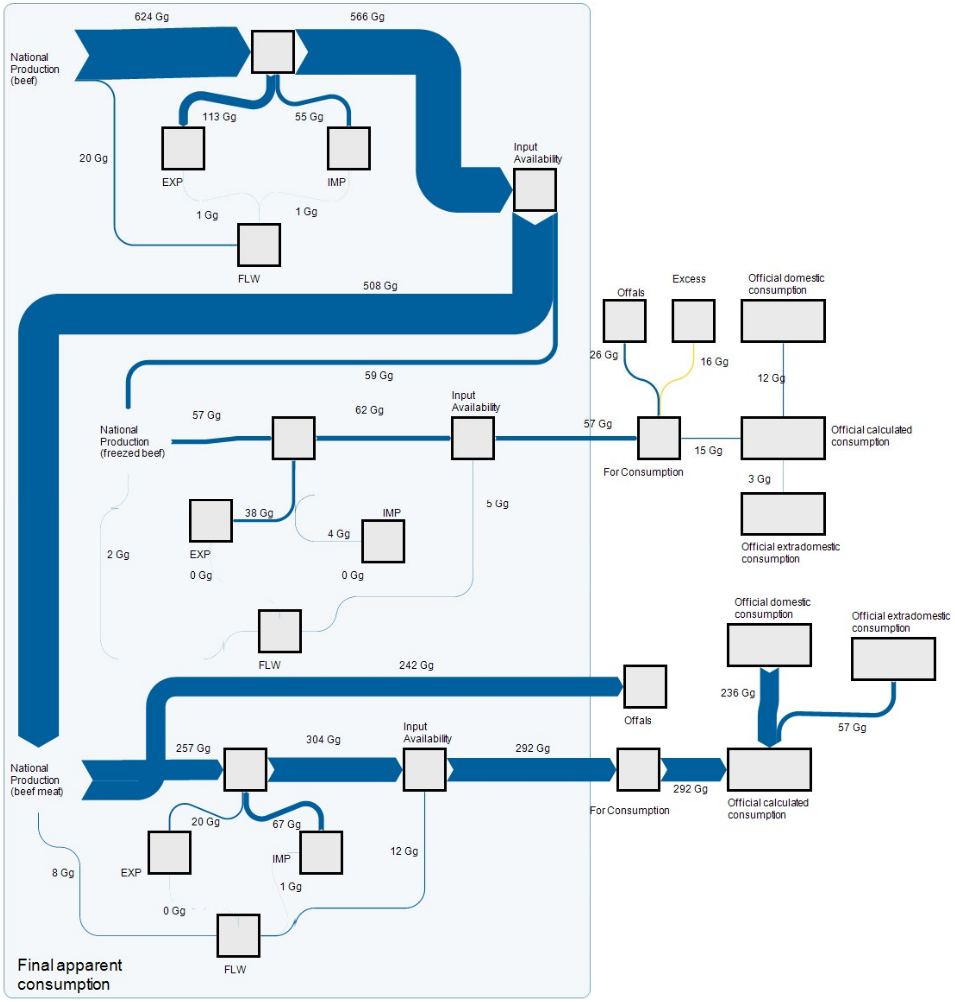

Figure S17 –Diagram of biomass flows for the beef food chain.

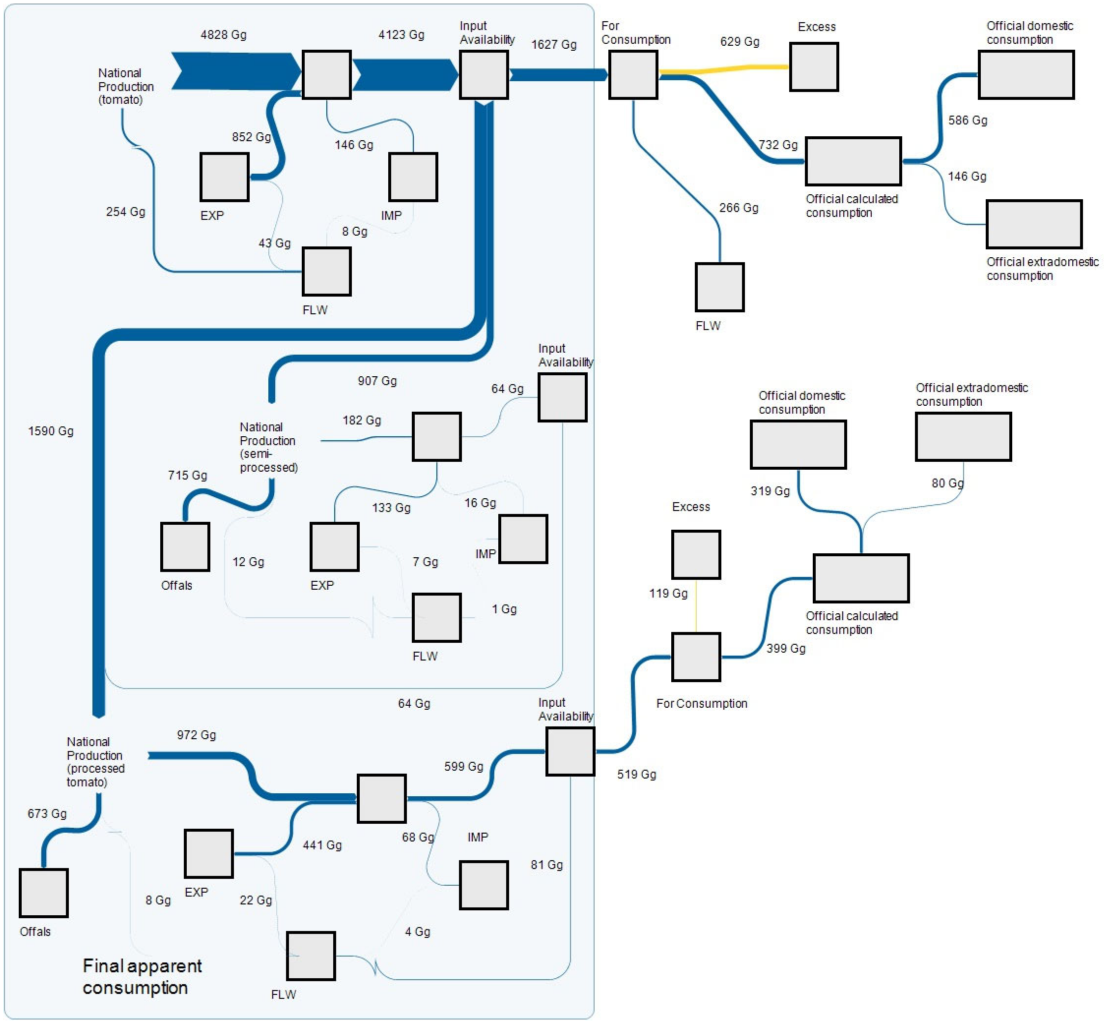

Figure S18 –Diagram of biomass flows for the tomato food chain.

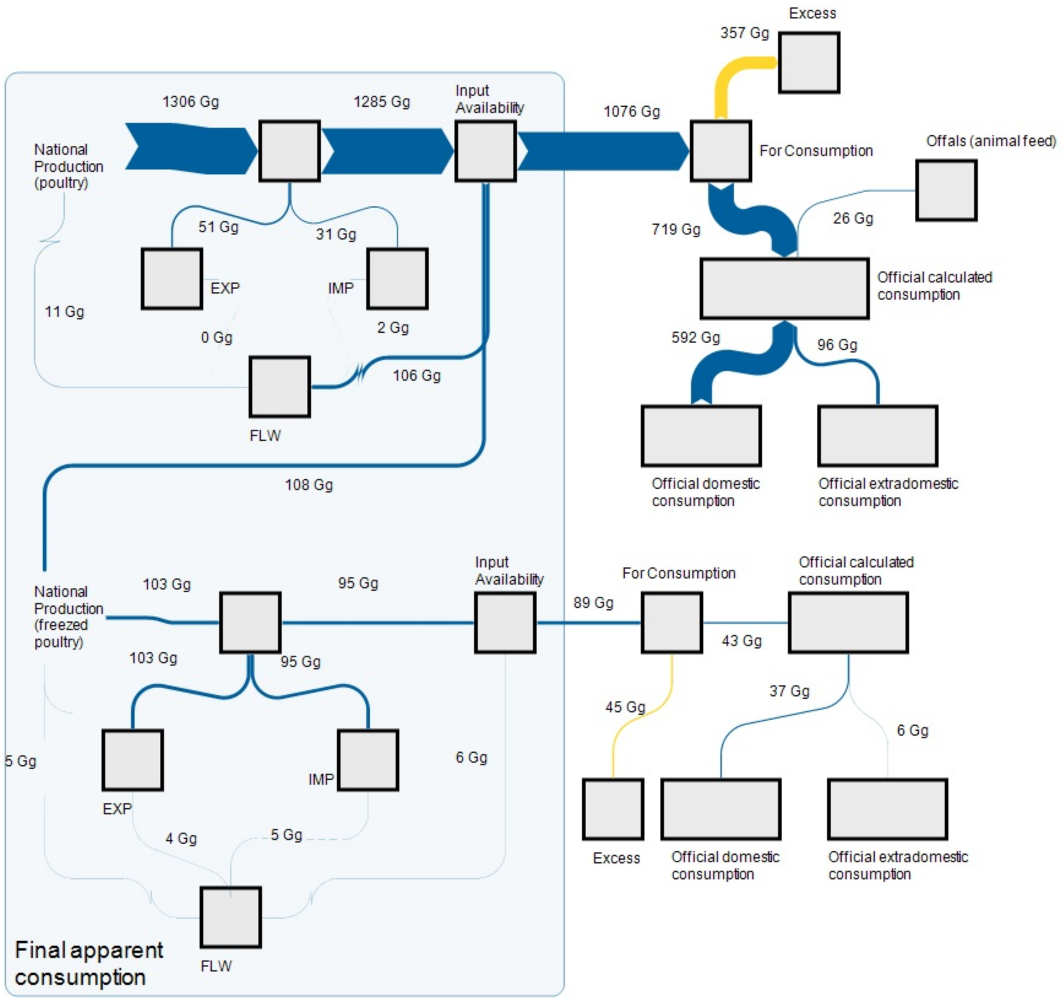

Figure S19 –Diagram of biomass flows for the chicken food chain.

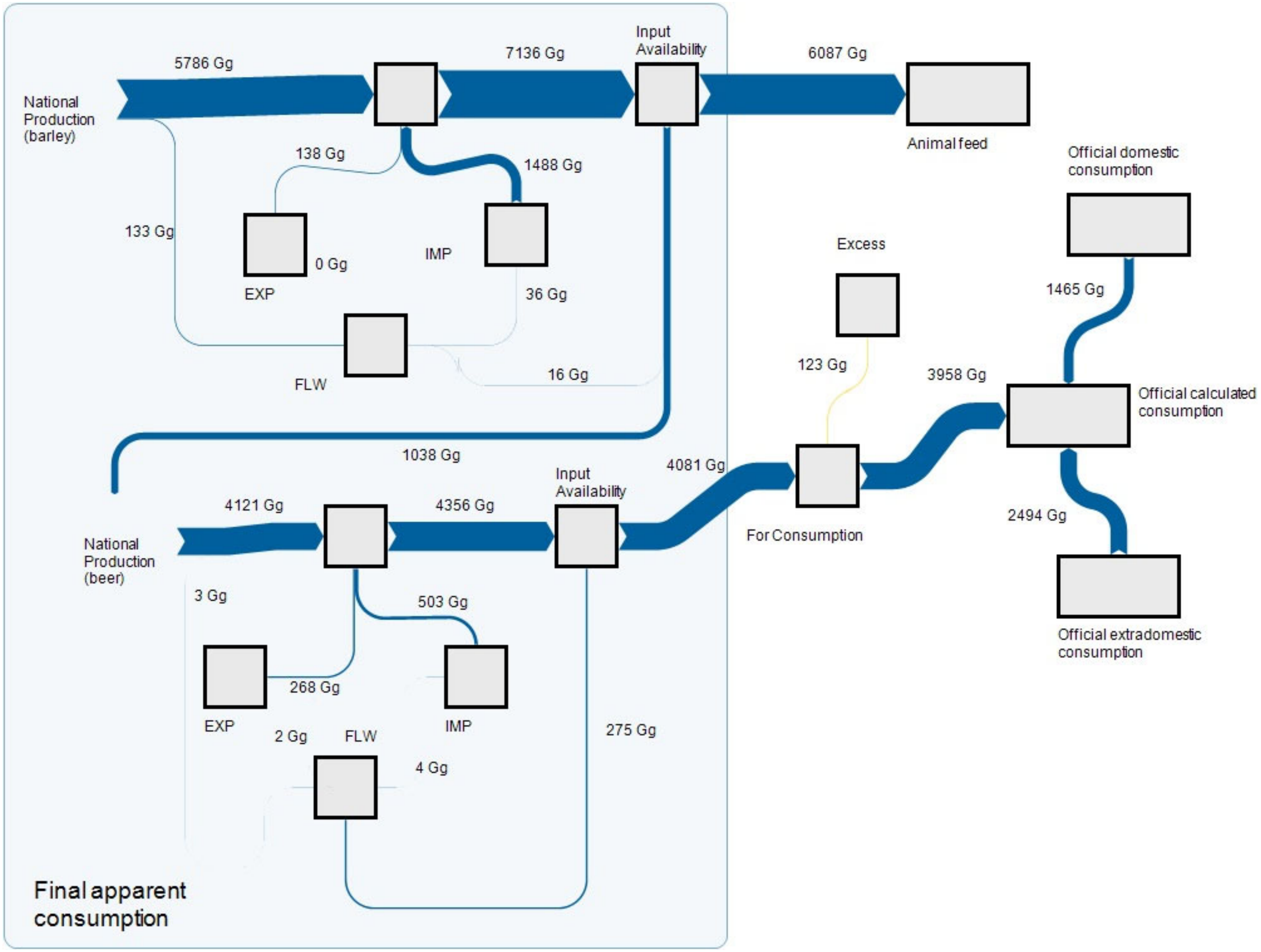

Figure S20 –Diagram of biomass flows for the beer food chain.

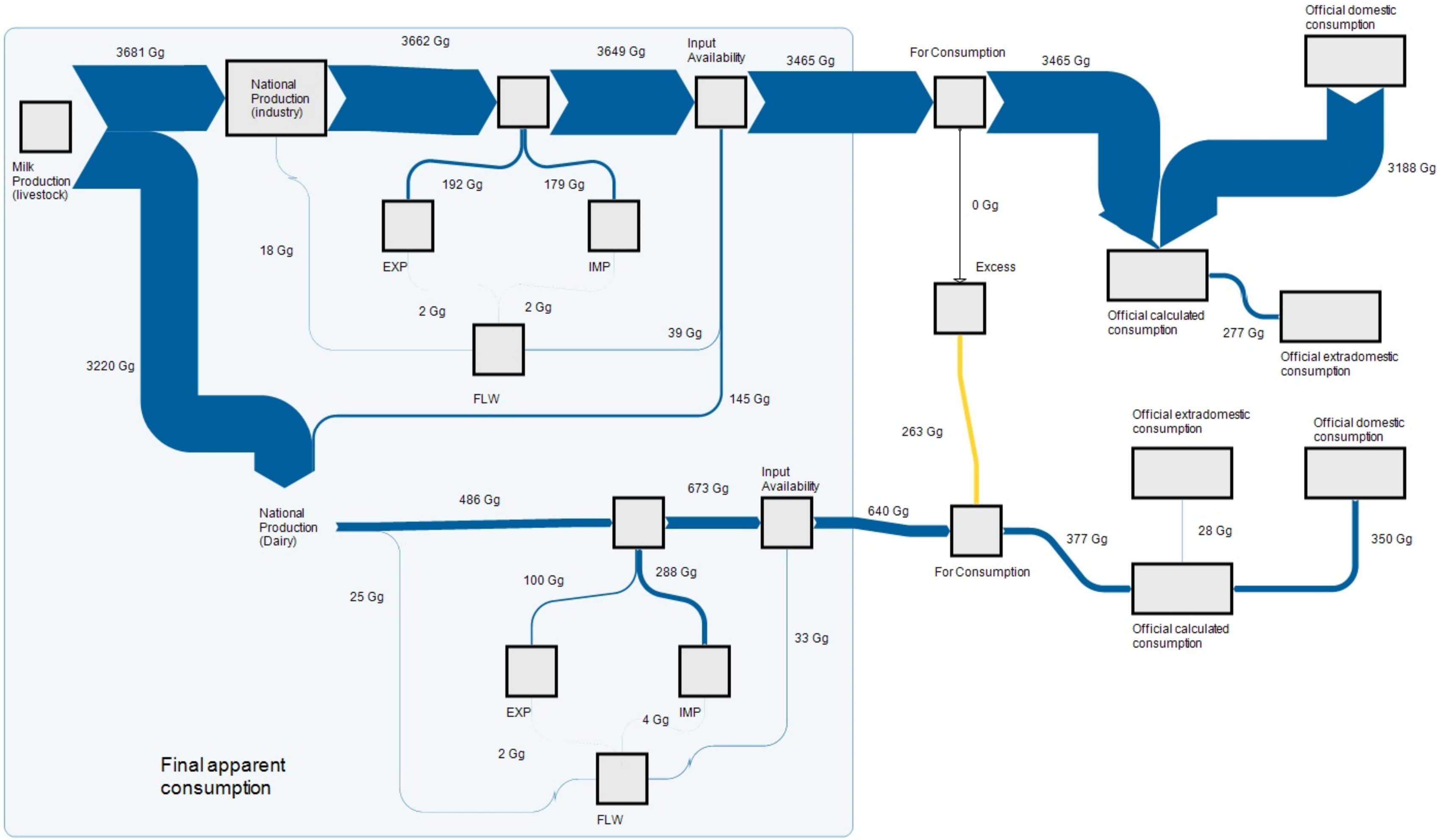

Figure S21 –Diagram of biomass flows for the milk food chain.

Table S2 indicates the weight of the ratio considered according to the type of food within the group of cereals and dairy products.

Table S2. Grams per serving of main foods belonging to the cereal and dairy groups.

Cereals	Serving (g)	Dairy	Serving (g)
Bread	50	Milk	225
Biscuits	40	Yogurt	125
Cereals	30	Cheese	95
Rice	60	Cream	50
Pasta	60	Ice cream	125
Potato	125	Dairy desserts	125

1. Statistics National Institute. Cuenta satélite del turismo en España. [Satellite account of tourism in Spain] 2017. Available online: https://www.ine.es/prensa/cst_2017.pdf (accessed on 24 March 2021).
2. Statistics National Institute. Encuesta Industrial de Producciones. [Industrial Production Survey]. 2017. Available online: https://www.ine.es/prensa/eiap_2017.pdf (accessed on 13 May 2020).
3. Johnstone, P.R.; Hartz, T.K.; LeStrange, M.; Nunez, J.J.; & Miyao, E.M. Managing fruit soluble solids with late-season deficit irrigation in drip-irrigated processing tomato production. HortScience, **2005**, *40*, 1857–1861. https://doi.org/10.21273/hortsci.40.6.1857.
4. Ciruelos Calvo, A.; de la Torre Carreras, R.; & González Ramos, C. Parámetros de calidad en el Tomate para industria. 2008. [Quality parameters in Tomato for industry]. Spanish. Badajoz: Universidad de Extremadura. Available online: https://www.unex.es/conoce-la-uex/centros/eia/archivos/iag/2007/2007_09%20Parametros%20de%20calidad%20en%20el%20tomate%20para%20industria.pdf (accessed on 22 June 2020).
5. Domínguez Pérez, I.; Rueda Castillo, I.J.; Pérez Vicente, A.; Vila Mompó, I.; Fayos Moltó, A.; Blanco Díaz, M.T.; Font Villa, R. *Parámetros de Calidad en Tomate Fresco. [Quality Parameters in Fresh Tomato]*; Consejería de Agricultura, Pesca y Desarrollo Rural, Instituto de Investigación y Formación Agraria y Pesquera: La Mojonera, Spain, 2012.
6. Lorente, J.; Valero, M.; de Ancos, B.; Martí, N.; García, S.; López, N.; Ramos, S.; Landajo, B.; Ferrer, J.; Alberdi, B. Capítulo: Aspectos industriales, en: El libro del zumo. In *Chapter: Industrial Aspects, in: The Juice Book*; de Zumos, A.E.d.F., Ed.; Agrícola Española: Valencia, España, 2011; pp. 79–116. (In Spanish).
7. Asselin-Balençon, A.; Broekema, R.; Teulon, H.; Gastaldi, G.; Houssier, J.; Moutia A; Rosseau V., Wermeille A. and Colomb V.. AGRIBALYSE v3.0: The French Agricultural and Food LCI Database. 2020. Available online: https://doc.agribalyse.fr/documentation-en/agribalyse-data/documentation (accessed on 24 July 2020).
8. Brodt, S.; Kramer, K.J.; Kendall, A.; & Feenstra, G. Comparing environmental impacts of regional and national-scale food supply chains: A case study of processed tomatoes. *Food Policy* **2013**, *42*, 106–114. https://doi.org/10.1016/j.foodpol.2013.07.004
9. Caldeira, C.; De Laurentiis, V.; Corrado, S.; van Holsteijn, F.; & Sala, S. Quantification of food waste per product group and along the food supply chain in the Europe Union: A Mass Flow Analysis. *Resour. Conserv. Recycl.* **2019**, *149*, 479–488. https://doi.org/10.1016/j.resconrec.2019.06.011.
10. Guzmán Casado, G.I.; Aguilera, E.; Soto, D.; Cid, A.; Infante Amate, J.; Garcia-Ruiz, R.; Herrera, A.; Villa, I.; de Molina, M.G.; *Methodology and Conversion Factors to Estimate the Net Primary Productivity of Historical and Contemporary Agroecosystems*; In Documentos de trabajo de la Sociedad Española de Historia Agraria (SEHA): Gerona, Spain no 1407; 2014.
11. Secondi, L.; Principato, L.; Ruini, L.; & Guidi, M. Reusing Food Waste in Food Manufacturing Companies: The Case of the Tomato-Sauce Supply Chain. *Sustainability* **2019**, *11*, 2154. https://doi.org/10.3390/su11072154
12. FAO. Global Food Losses and Food Waste. Extent, Causes and Prevention. 2011, Rome. Available online: https://www.fao.org/3/i2697e/i2697e.pdf (accessed on 12 February 2020).
13. Hellweg, Stefanie, and Rainer Zah. 'What Is New at the Data Front?' *International Journal of Life Cycle Assessment* **2016,** 21 (9): 1215–1217. https://doi.org/10.1007/s11367-016-1126-5.
14. Eurostat. PRODCOM. Lista 2019. EUROSTAT. Available online: https://ec.europa.eu/eurostat/ramon/nomenclatures/index.cfm?TargetUrl=LST_NOM&StrGroupCode=CLASSIFIC&StrLanguageCode=EN&IntFamilyCode=&TxtSearch=prodcom&IntCurrentPage=1 (accessed on 16 June 2020).
